# Analysis of Arsenite Acute Toxic Effects Based on Bayesian Model Averaging in Embryo, Larva, Juvenile and Adult Zebrafish

**DOI:** 10.3390/toxics14100840

**Published:** 2026-09-22

**Authors:** Yuanhui Zhu, Yan Zhang, Tingxu Jin, Shougang Zhang, Guixiang Ji

**Affiliations:** 1 Nanjing Center for Disease Control and Prevention, Nanjing 210003, China; zbzyhcom@163.com (Y.Z.); zhangyan871207@163.com (Y.Z.); 2 School of Public Health, Guizhou Medical University, Guiyang 561113, China; jintingxu@gmc.edu.cn; 3 Nanjing Institute of Environmental Sciences, Ministry of Ecology and Environment, Nanjing 210042, China

**Keywords:** arsenic, zebrafish in different life stages, survival rate, Bayesian Benchmark Dose

## Abstract

Arsenic is a ubiquitous environmental pollutant with well-documented human health risks, among which trivalent arsenic (arsenite) exhibits the highest toxicity. Although the acute toxicity of arsenite has been extensively studied in zebrafish embryos, its effects across other developmental stages remain largely unknown. In the present study, the acute toxicity of arsenite was evaluated across four zebrafish life stages—embryo, larva, juvenile, and adult—using both probit regression and Bayesian Model Averaging (BMA). Both methods revealed that LC_50_ values progressively decreased with increasing exposure time across all life stages, and susceptibility followed the order of embryo < larva < juvenile < adult. Additionally, Benchmark Dose (BMD), Benchmark Dose Lower Bound (BMDL), and Benchmark Dose Upper Bound (BMDU) values decreased with prolonged exposure, confirming a clear dose–response relationship. Notably, LC_50_ values estimated by BMA were consistently higher than those derived from probit regression, suggesting that the conventional approach may produce conservative estimates. Our findings not only establish a robust foundation for arsenic risk assessment across the entire zebrafish life cycle, but also provide experimental evidence that calls for a life-stage-informed revision of current arsenic safety thresholds.

## 1. Introduction

Arsenic is the 20th most abundant element in the Earth’s crust and is ubiquitously distributed throughout the natural environment (air, water and soil) with highly toxic effects [1,2]. Arsenic occurrence in the environment is primarily derived from geogenic processes, such as mineral weathering and volcanic emissions, while anthropogenic activities, such as mining, nonferrous metal smelting, coal combustion, and the use of arsenic-based pesticides, further amplify its distribution [3,4]. In nature, arsenic predominantly exists in two valence states: trivalent arsenic (As(III)) and pentavalent arsenic (As(V)) [3]. Trivalent arsenic is 30–60 times more toxic than its pentavalent counterpart, primarily due to its higher affinity for thiol (–SH) groups in proteins and enzymes, as well as its enhanced cellular uptake capacity [5]. Consequently, several animal- and cell-based studies have been conducted using sodium arsenite (NaAsO_2_), a representative trivalent arsenic compound, over the past few decades [6,7,8].

Previous research has shown that arsenic primarily enters the human body through drinking water or contaminated food and can cause various diseases, including cancers (such as skin, lung, and bladder cancer), cardiovascular disorders, non-alcoholic fatty liver disease and type 2 diabetes [9,10,11,12]. In addition, arsenic present in aquatic environments exerts broad impacts on aquatic organisms and undergoes bioaccumulation along the food chain, thereby threatening the integrity of aquatic ecosystems [13]. Therefore, in 1984, the World Health Organization (WHO) first set a guideline value of 10 μg/L for arsenic in drinking water [14]. Yet, more recent work suggests that between 94 and 220 million people across the globe (with 94% located in Asia) face groundwater arsenic concentrations surpassing this safety benchmark [9]. For example, the work by Dipankar et al. reported that the Ganga River basin suffers from heightened arsenic contamination, reaching 4730 µg/L in groundwater, roughly 1000 µg/L in irrigation water, and as high as 3947 µg/kg in foodstuffs [15].

Based on the pervasiveness of arsenic in the environment and its ecological threats, there is a pressing need to elucidate its impacts on aquatic organisms and ecosystem health. As an in vitro fertilization model organism, zebrafish (*Danio rerio*) is particularly suitable for aquatic ecotoxicology research on contaminant exposure due to its high fecundity, rapid development, and sensitivity to a wide range of contaminants [16]. The median lethal concentration (LC_50_) value, used to assess the survival rates, is a core quantitative indicator in acute toxicity testing for assessing the aquatic ecotoxicity of aquatic environmental pollutants [17]. In aquatic environments, fish are simultaneously exposed to contaminants at various life stages (embryo, larval, juvenile, and adult). Therefore, assessing the toxic effects of a contaminant on all life stages can provide more comprehensive toxicity data, thereby avoiding the one-sidedness of data derived from a single life stage.

Multiple methods are available for calculating the LC_50_, such as the Litchfield–Wilcoxon method, Karber’s approach, and regression analysis, all of which are grounded in the frequentist statistical framework [18,19,20]. Nevertheless, each method is subject to specific constraints. The Karber method is valid only when the response variable adheres to a normal distribution [18]; regression analysis requires careful evaluation of model goodness-of-fit [19], while the Litchfield–Wilcoxon method relies on manually fitted lines on graph paper, a subjective procedure that often produces variable estimates [20]. Unlike frequentist statistics, which rely on confidence levels interpreted under hypothetical repetition, Bayesian methods assign probability distributions to unknown parameters to better reflect scientific uncertainty [21]. Hence, in 2022, the European Food Safety Authority (EFSA) advocated for a paradigm shift from traditional frequentist methods to Bayesian approaches in risk assessment [22]. In Bayesian approaches, the Benchmark Dose (BMD), Benchmark Dose Lower Bound (BMDL) and Benchmark Dose Upper Bound (BMDU) are increasingly preferred over the traditional no observed adverse effect level (NOAEL), as they make fuller use of the dose–response data and provide a more precise reference point for toxicity threshold estimation [21]. Specifically, the Bayesian Benchmark Dose (BBMD) methodology applies the Bayesian Model Averaging (BMA) framework to comprehensively characterize the dose–response relationship. The BMA framework integrates eight distinct mathematical models, comprising a set of candidate models that are each weighted by their posterior probability, thereby avoiding the limitations of selecting a single “best-fit” model [23,24,25]. Our research group has previously used the BMA framework to study the environmental exposure to arsenic in early-life-stage zebrafish [26]. However, a notable paucity of research exists regarding the comparative toxicity of arsenic across different developmental stages in zebrafish. To address this gap, the present study employed the conventional acute toxicity metric, the LC_50_, to elucidate and quantify the toxicological effects of arsenic exposure throughout these critical life stages.

In summary, although arsenic toxicity has been studied in zebrafish at the selected developmental life stages, a systematic comparison of its acute effects across the entire life cycle—from embryo to adult—remains lacking. To address this gap, the present study was designed to (i) systematically assess the LC_50_ value of arsenite in zebrafish embryos, larvae, juveniles, and adults under consistent exposure conditions; (ii) apply the BMA framework to derive BMD, BMDL and BMDU for multiple toxic endpoints across all life stages, thereby overcoming the limitations of conventional frequentist methods; and (iii) identify the sensitive life stage of zebrafish to arsenic exposure. This work establishes a stage-specific toxicity profile of arsenic across four key zebrafish life stages, aiming to provide a more robust and comparable toxicological foundation for aquatic arsenic ecological risk assessment, with direct applicability to the development of science-based protective exposure thresholds.

## 2. Materials and Methods

### 2.1. Chemicals and Reagents

NaAsO_2_ (99% purity) was obtained from Merck Drugs & Biotechnology, Darmstadt, Germany. To ensure concentration stability in later experiments, NaAsO_2_ was kept at −20 °C, and working solutions were freshly prepared immediately before each use.

### 2.2. Environmental Conditions and Arsenic Exposure for Zebrafish Experiments

Wild-type zebrafish (TU strain in adult) were sourced from the Institute of Hydrobiology, Chinese Academy of Sciences (Wuhan, China). All experiments in this study adhered to China’s Guidelines for the Care and Use of Experimental Animals and the animal welfare guidelines of the Nanjing Institute of Environmental Sciences (approval number: IACUC-20220910). Adult zebrafish were kept in optimal conditions and received a diet of brine shrimp three times daily. The aquatic condition of zebrafish was consistently held at 28.0 ± 0.5 °C. Prior to the experimental procedure, sexually mature zebrafish in a ratio of four males to two females were placed overnight in a breeding tank fitted with a partition. Spawning was triggered at 6:30 a.m. the next morning, coinciding with the beginning of the light phase. Developing embryos were then chosen and randomly distributed into 10 cm diameter glass dishes for later experimental use.

### 2.3. Survival Rate Test in Embryo, Larva, Juvenile and Adult Zebrafish

To systematically evaluate the acute toxic effects induced by NaAsO_2_ across different life stages of zebrafish, independent stage-specific exposure systems were established for four distinct developmental life stages. For the embryonic stage, NaAsO_2_ exposure was initiated at 4 h post-fertilization (hpf), using a concentration gradient of 0, 50, 100, 200, 250, 300, 350, 360, 375, 400, 600, 800, 1000, and 1200 mg/L. For the larval stage, NaAsO_2_ exposure commenced at 5 days post-fertilization (dpf) with a concentration series of 0, 10, 50, 100, 150 and 200 mg/L. For the juvenile stage, NaAsO_2_ exposure was started at 30 dpf, covering concentrations of 0, 25, 50, 55, 60, 65, 70, 75 and 100 mg/L. For the adult stage (120 dpf and older), NaAsO_2_ exposure was initiated using a concentration gradient of 0, 20, 30, 40, 45, 50, 55, 60, 65 and 70 mg/L. For the embryonic and larval zebrafish groups, test organisms were maintained in 6-well culture plates, with 10 embryos or larvae allocated per well and three replicate wells set up for each exposure concentration. For the juvenile and adult zebrafish groups, three parallel 5 L plastic tanks were assigned to every concentration, with 10 test zebrafish maintained in each tank. The NaAsO_2_ exposure solutions for all experimental groups were renewed at a 50% volume exchange rate daily, and any dead test organisms were removed daily. The survival rates of zebrafish at the embryonic, larval, juvenile and adult life stages were documented at 24, 48, 72 and 96 h post-initiation of NaAsO_2_ exposure, in accordance with OECD Test 236 and OECD Test 210 [17,27]. At the end of the experiment, surviving zebrafish were humanely euthanized with an overdose of MS-222 (tricaine methanesulfonate) to minimize pain and suffering, followed by confirmation of death.

### 2.4. LC_50_, BMD and BMDL Calculation

Survival rate indicators were analyzed using the Bayesian Benchmark Dose Analysis System (BBMD) (https://benchmarkdose.org). A Markov Chain Monte Carlo (MCMC) simulation was implemented in BBMD to perform Bayesian inference. After that, LC_50_, BMD and BMDL values were calculated using eight Bayesian models in the BBMD system. Define Rhat for 1–1.05, and the posterior predictive *p* (PPP) value for 0.05–0.95. The LC_50_ values were calculated via the probit regression model implemented in SPSS 25.0, as well as using the eight Bayesian models provided in the BBMD system. BMD and BMDL values for eight Bayesian models after NaAsO_2_ exposure were calculated at 10% BMR. After that, experimental data from each indicator were used to determine the weights assigned to eight Bayesian models. LC_50_, BMD and BMDL values of BMA model were calculated by the weighted average of the eight Bayesian models for each set of experimental data.

### 2.5. Statistical Analysis

Statistical data analysis was conducted using SPSS software (version 25.0). All datasets were initially assessed for homogeneity of variance. Following analysis of variance (ANOVA), pairwise comparisons of group means were performed using the Student–Newman–Keuls (SNK) method when variances were homogeneous; for data with heterogeneous variances, Tamhane’s T2 test was applied. Results are expressed as mean ± standard error of the mean (SEM). Statistical significance was defined as a *p*-value of less than 0.05.

## 3. Results

### 3.1. LC_50_ Analyses by the Probit Regression Model of Embryo, Larva, Juvenile and Adult Zebrafish After NaAsO_2_ Exposure

LC_50_ values in zebrafish at four life stages after NaAsO_2_ exposure were calculated by the probit regression model using SPSS 25.0. As a result, the LC_50_ values of zebrafish embryo after 24 h, 48 h, 72 h and 96 h NaAsO_2_ exposure were 1063.39 mg/L, 678.72 mg/L, 349.76 mg/L, and 204.44 mg/L, respectively (Figure 1A). The LC_50_ values of larva zebrafish after 24 h, 48 h, 72 h and 96 h NaAsO_2_ treatment were 133.88 mg/L, 109.68 mg/L, 101.76 mg/L, and 93.33 mg/L, respectively (Figure 1B). The LC_50_ values of juvenile zebrafish after 24 h, 48 h, 72 h and 96 h NaAsO_2_ exposure were 71.15 mg/L, 70.20 mg/L, 69.89 mg/L, and 66.00 mg/L, respectively (Figure 1C). The LC_50_ values of adult zebrafish after 24 h, 48 h, 72 h and 96 h NaAsO_2_ treatment were 61.66 mg/L, 57.17 mg/L, 54.18 mg/L, and 52.95 mg/L, respectively (Figure 1D). Collectively, the above results indicated that the LC_50_ values for zebrafish within the same life stage gradually decreased after NaAsO_2_ exposure. Furthermore, susceptibility to arsenite followed the order: embryo < larva < juvenile < adult, with adults being most sensitive.

### 3.2. LC_50_, BMD, BMDL and BMDU Analyses of Zebrafish Embryo After NaAsO_2_ Exposure in BMA and Eight Bayesian Models

The BBMD method was used to calculate the values of LC_50_, BMD, BMDL and BMDU after NaAsO_2_ exposure in zebrafish embryo. As shown in Table 1, in the BMA model, the LC_50_ value of NaAsO_2_ in zebrafish embryo declined progressively with exposure time, establishing a well-defined time–effect relationship. Across the four sequential NaAsO_2_ exposure time points, the benchmark dose metrics (BMD, BMDL and BMDU) exhibited varying dynamic trends, with the lowest overall values for each indicator consistently recorded at the last exposure stage, showing a significant decrease relative to the preceding time periods. In addition, the posterior probability density of the BMD based on BMA in zebrafish embryo after NaAsO_2_ exposure was depicted in Figure 2. Specific LC_50_, BMD, BMDL and BMDU values of eight single Bayesian models were shown in Supplementary Tables S1–S4. Supplementary Figures S1 and S2 depicted the posterior probability density of the BMD based on eight BMD models, and the dose–response relationship for eight BMD models in zebrafish embryo after NaAsO_2_ exposure, respectively.

### 3.3. LC_50_, BMD, BMDL and BMDU Analyses of Larval Zebrafish After NaAsO_2_ Exposure in BMA and Eight Bayesian Models

As shown in Table 2, in BMA model, the LC_50_ and BMDU values of NaAsO_2_ in larval zebrafish decreased progressively with the extension of exposure time, establishing a clear time–effect relationship. For BMD and BMDL, an overall increasing trend was observable with the increase in NaAsO_2_ exposure time. In addition, the posterior probability density of the BMD based on BMA in larva zebrafish after NaAsO_2_ exposure was depicted in Figure 3. Specific LC_50_, BMD, BMDL and BMDU values of eight single Bayesian models were shown in Supplementary Tables S5–S8. Supplementary Figures S3 and S4 depicted the posterior probability density of the BMD based on eight BMD models, and the dose–response relationship for eight BMD models in larval zebrafish after NaAsO_2_ exposure, respectively.

### 3.4. LC_50_, BMD, BMDL and BMDU Analyses of Juvenile Zebrafish After NaAsO_2_ Exposure in BMA and Eight Bayesian Models

The acute toxicity endpoints yielded by BMA modeling revealed a clear time-dependent pattern. The LC_50_ of NaAsO_2_ in juvenile zebrafish declined progressively with exposure time, establishing a well-defined time–effect relationship. Meanwhile, BMD and BMDU values all displayed a general downward trend as the NaAsO_2_ exposure time increased. Across the full 96 h NaAsO_2_ exposure period, the BMDL values demonstrated an overall rising trend with the extension of exposure time (Table 3). In addition, the posterior probability density of the BMD based on BMA in juvenile zebrafish after NaAsO_2_ exposure was depicted in Figure 4. Specific LC_50_, BMD, BMDL and BMDU values of eight single Bayesian models were shown in Supplementary Tables S9–S12. Supplementary Figures S5 and S6 depicted the posterior probability density of the BMD based on eight BMD models, and the dose–response relationship for eight BMD models in juvenile zebrafish after NaAsO_2_ exposure, respectively.

### 3.5. LC_50_, BMD, BMDL and BMDU Analyses of Adult Zebrafish After NaAsO_2_ Exposure in BMA and Eight Bayesian Models

The acute toxicity endpoints calculated by BMA demonstrated clear time-dependent characteristics in adult zebrafish: LC_50_ and BMDL values decreased steadily from 24 h to 96 h of NaAsO_2_ exposure, forming a robust time–effect relationship. For BMD and BMDU, an overall decreasing trend was still observable with increasing NaAsO_2_ exposure time (Table 4). In addition, the posterior probability density of the BMD based on BMA in adult zebrafish after NaAsO_2_ exposure was depicted in Figure 5. Specific LC_50_, BMD, BMDL and BMDU values of eight single Bayesian models were shown in Supplementary Tables S13–S16. Supplementary Figures S7 and S8 depicted the posterior probability density of the BMD based on eight BMD models, and the dose–response relationship for eight BMD models in adult zebrafish after NaAsO_2_ exposure, respectively.

## 4. Discussion

Acute toxicity indices such as the LC_50_ can provide critical support for long-term toxicity experiments and serve as a basis for future revisions of relevant aquatic quality standards [21]. Due to the widespread distribution of arsenic in the aquatic environment, previous studies have reported a variety of toxic effects in zebrafish after arsenic treatment. A previous study has shown that the survival rate of zebrafish embryo at 120 hpf is approximately 10% after exposure to 2.0 mM NaAsO_2_ [28]. Sun et al. reported that the LC_50_ value of NaAsO_2_ for adult zebrafish was 42 mg/L [29]. Unlike most previous studies that typically focus on a single life stage of zebrafish, we selected one time point for acute toxicity testing at each of the four life stages, thus comprehensively revealing the aquatic ecotoxic effects of arsenic on zebrafish.

Our results demonstrated that, following arsenic exposure, the LC_50_ values of zebrafish followed the order: adult < juvenile < larva < embryo, indicating that adult zebrafish are the most sensitive to arsenic exposure. Similar to our current research, the study by Shabnam Ansari also verified that the LC_50_ values of larva zebrafish were lower than those in embryos for three heavy metals [30]. Results verified that the LC_50_ value of Zn in zebrafish embryo was 41.08 mg/L after 72 h exposure, while the value in larva zebrafish was 20.16 mg/L; the LC_50_ value of Ni in zebrafish embryo was 69.78 mg/L after 72 h exposure, while the value in larva zebrafish was 35.96 mg/L; the LC_50_ value of Cr in zebrafish embryo was 15.99 mg/L after 72 h exposure, while the value in larva zebrafish was 8.73 mg/L [30]. Another study also showed that zebrafish embryos are less sensitive to hexavalent chromium (Cr^6+^) than adults, with 96 h LC_50_ values of 145.7 mg/L for embryos and 39.4 mg/L for adults. The authors speculated that this difference might be due to the chorion acting as a physical barrier, delaying the entry of the pollutant into the embryo [31]. However, the authors also showed that neurobiomarker responses during the embryonic stage were much more sensitive than those in adults, manifesting as stronger neurotoxicity, including inhibition of cholinesterase and glutathione S-transferase activities [31].

It is worth mentioning that, except for some LC_50_ values derived from larval zebrafish, the LC_50_ values which were calculated using the BMA method were consistently higher than those obtained via probit regression analysis. This discrepancy may be attributed to the relatively wide concentration gradients used in the acute toxicity tests for larval zebrafish compared to the other three life stages, which likely reduced the accuracy of model fitting. Our findings also reveal that probit regression tends to yield conservative LC_50_ estimates, highlighting BMA as a more robust and precise alternative for future ecotoxicological studies.

At present, the BMDL value has gradually become a preferred alternative to NOAEL in recent toxicology research [32]. In traditional toxicology, NOAEL is defined as the highest tested concentration that does not show a statistically significant difference from the control group (i.e., an actual experimental value). Consequently, its reliability heavily depends on the concentrations selected in the experimental design. In contrast, BMD analysis evaluates data by accounting for specific characteristics of the dataset (e.g., data dispersion and dose–response information). In addition, BMDL represents the lower bound of the 95% confidence interval of the BMD value; hence, the data constraints such as sample size are less critical than for NOAEL [32]. Researchers have verified that the Bayesian method is more sensitive than the traditional NOAEL method [33]. In 2017, the European Food Safety Authority (EFSA) showed that, compared to using a single BMD model, the BMA approach, which enables multi-model estimation and inference, is the optimal method for addressing model uncertainty while simultaneously handling uncertainties related to sampling error in the data [34].

Unlike mammalian models, zebrafish currently lack a well-established and widely accepted framework for cross-species risk extrapolation to humans, which limits their application in drug safety assessment. Nevertheless, zebrafish experiments are generally considered more aligned with the 3R principles (Replacement, Reduction, Refinement) of toxicology than mammalian experiments, offering ethical and practical advantages [35]. Recent efforts have begun to address the extrapolation gap; for example, Huante Bio (in China) proposed a patent (ZL 2020 1 0256146.8) that derives a conversion formula based on the NOAEL method to estimate human equivalent doses from zebrafish data. Although such an approach represents a promising step, it still requires further validation. Therefore, the BMA method could be employed to obtain more robust and precise safety evaluation data in future studies.

## 5. Conclusions

Taken together, the present study systematically quantified the acute toxic effects of NaAsO_2_ across four life stages of zebrafish, providing a full set of LC_50_, BMD, BMDL and BMDU values derived from both probit regression and BMA methods. Both the conventional probit regression model and the BMA approach indicated that the LC_50_ value for zebrafish progressively decreased with increasing NaAsO_2_ exposure time across all life stages. In addition, susceptibility to NaAsO_2_ followed the order: embryo < larva < juvenile < adult (with adults being the most sensitive). Our results also indicate that, with prolonged NaAsO_2_ exposure, the BMD, BMDL, and BMDU values for zebrafish at four life stages gradually decreased, exhibiting a dose–response relationship. Meanwhile, the LC_50_ values estimated by BMA were generally higher than those derived from probit regression. Our results reveal that probit regression tends to yield conservative LC_50_ estimates, highlighting BMA as a more robust and precise alternative for future ecotoxicological studies. Our findings not only establish a robust foundation for arsenic risk assessment across the entire zebrafish life cycle, but also provide experimental evidence that calls for a life-stage-informed revision of current arsenic safety thresholds.

## Figures and Tables

**Figure 1:**
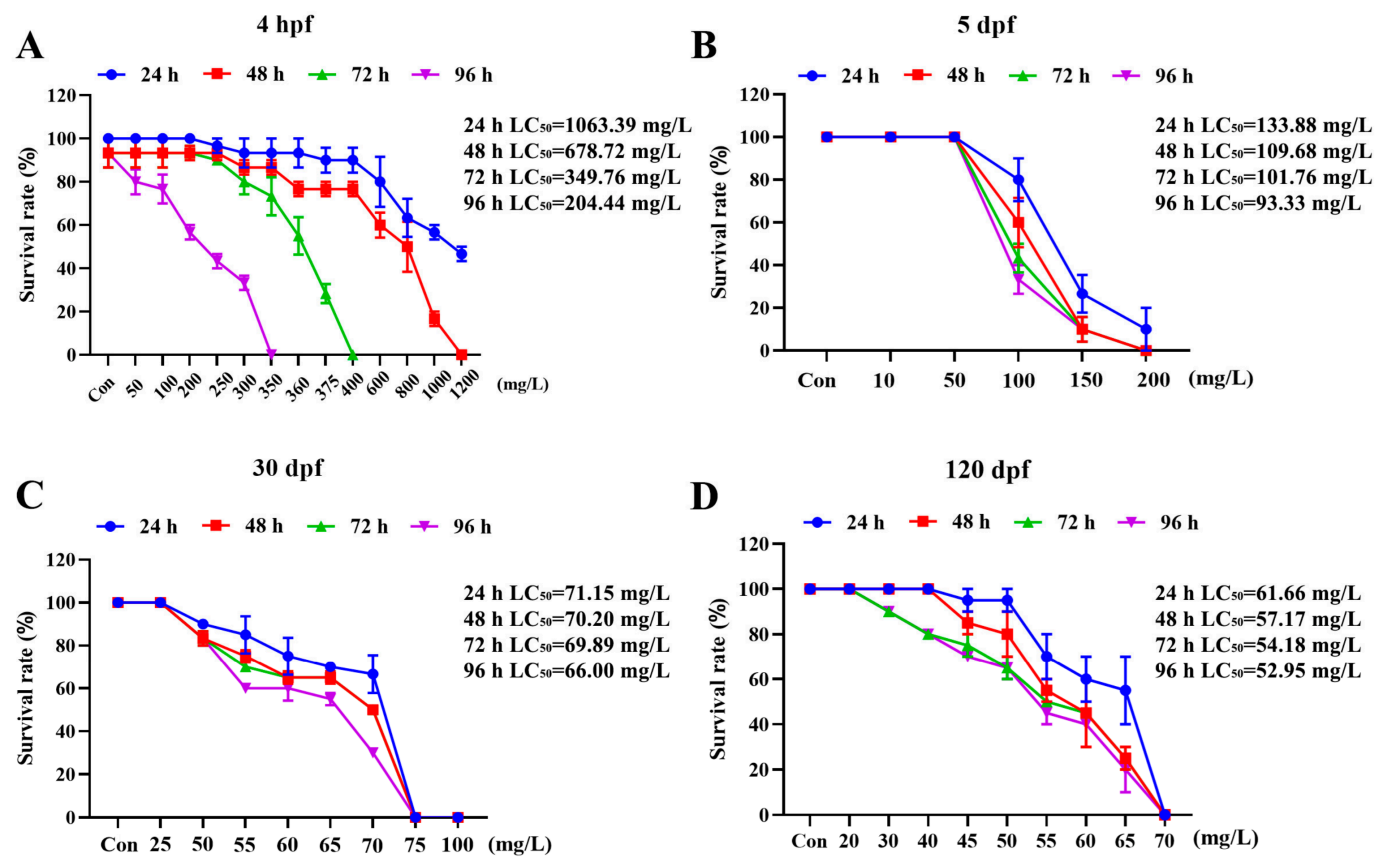
LC_50_ values calculated by the probit regression model of embryo, larva, juvenile and adult zebrafish after 24 h, 48 h, 72 h and 96 h NaAsO_2_ exposure. (**A**) LC_50_ values of zebrafish embryo (4 hpf) after 24 h, 48 h, 72 h and 96 h NaAsO_2_ exposure. (**B**) LC_50_ values of larva zebrafish (5 dpf) after 24 h, 48 h, 72 h and 96 h NaAsO_2_ exposure. (**C**) LC_50_ values of juvenile zebrafish (30 dpf) after 24 h, 48 h, 72 h and 96 h NaAsO_2_ exposure. (**D**) LC_50_ values of adult zebrafish (120 dpf) after 24 h, 48 h, 72 h and 96 h NaAsO_2_ exposure.

**Figure 2:**
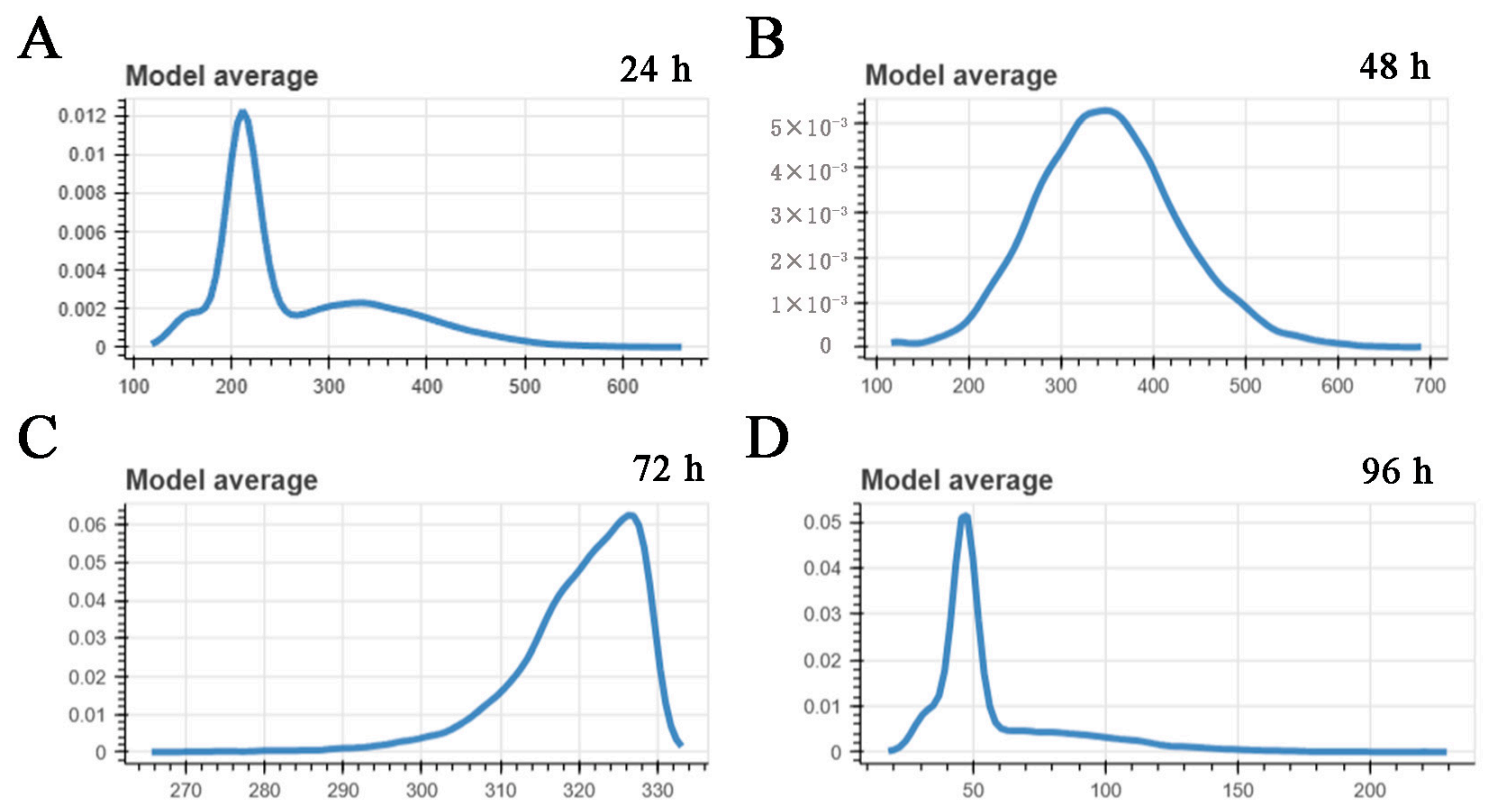
Posterior probability density of the BMD based on BMA in zebrafish embryo after NaAsO_2_ exposure. (**A**) Posterior probability density of the BMD based on BMA in zebrafish embryo after 24 h NaAsO_2_ exposure. (**B**) Posterior probability density of the BMD based on BMA in zebrafish embryo after 48 h NaAsO_2_ exposure. (**C**) Posterior probability density of the BMD based on BMA in zebrafish embryo after 72 h NaAsO_2_ exposure. (**D**) Posterior probability density of the BMD based on BMA in zebrafish embryo after 96 h NaAsO_2_ exposure.

**Figure 3:**
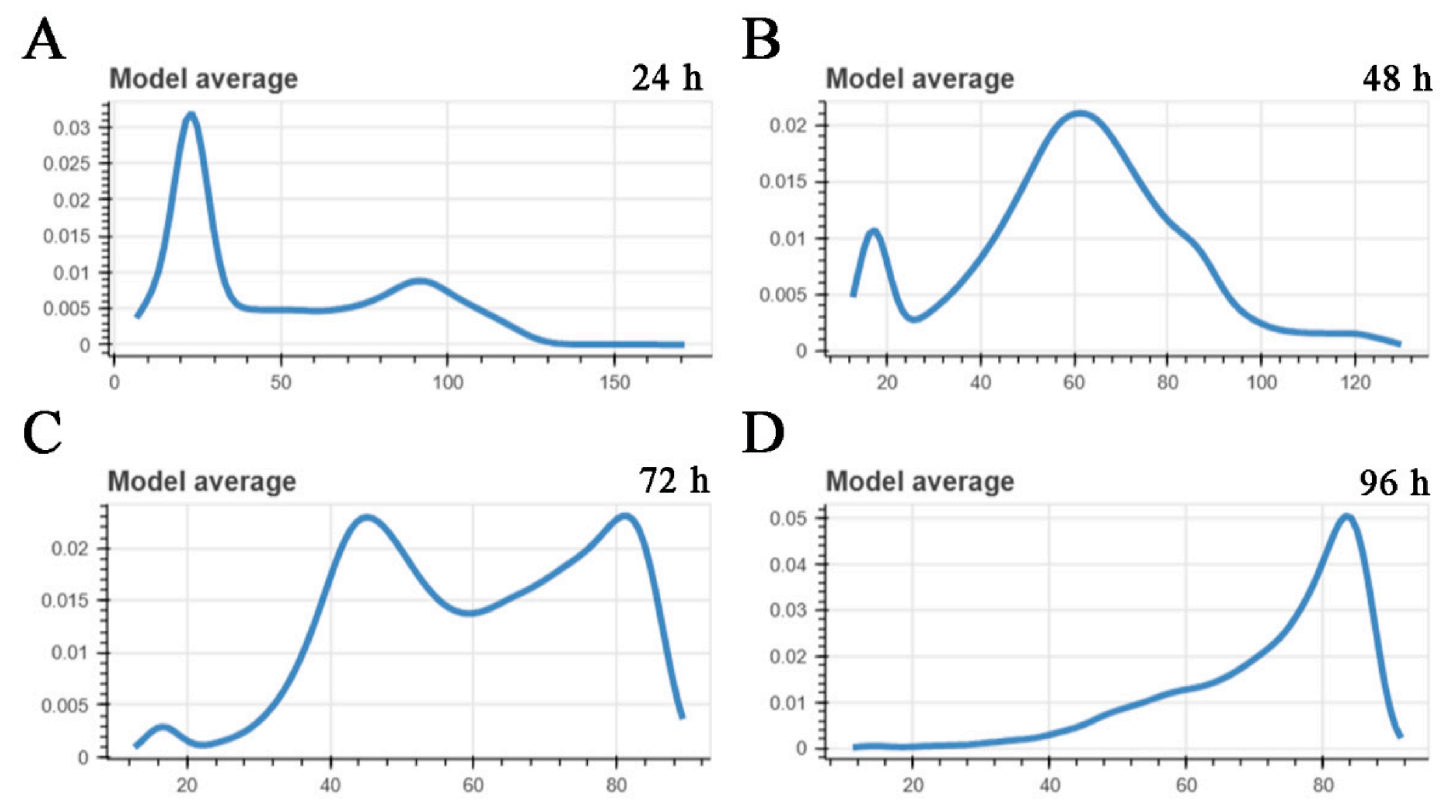
Posterior probability density of the BMD based on BMA in larva zebrafish after NaAsO_2_ exposure. (**A**) Posterior probability density of the BMD based on BMA in larva zebrafish after 24 h NaAsO_2_ exposure. (**B**) Posterior probability density of the BMD based on BMA in larva zebrafish after 48 h NaAsO_2_ exposure. (**C**) Posterior probability density of the BMD based on BMA in larva zebrafish after 72 h NaAsO_2_ exposure. (**D**) Posterior probability density of the BMD based on BMA in larva zebrafish after 96 h NaAsO_2_ exposure.

**Figure 4:**
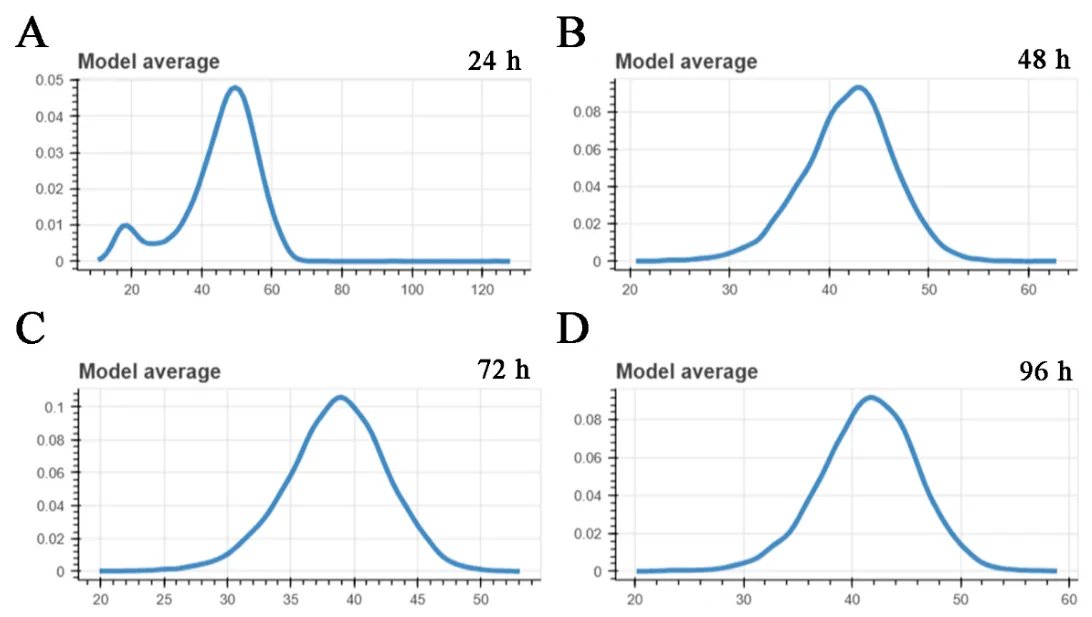
Posterior probability density of the BMD based on BMA in juvenile zebrafish after NaAsO_2_ exposure. (**A**) Posterior probability density of the BMD based on BMA in juvenile zebrafish after 24 h NaAsO_2_ exposure. (**B**) Posterior probability density of the BMD based on BMA in juvenile zebrafish after 48 h NaAsO_2_ exposure. (**C**) Posterior probability density of the BMD based on BMA in juvenile zebrafish after 72 h NaAsO_2_ exposure. (**D**) Posterior probability density of the BMD based on BMA in juvenile zebrafish after 96 h NaAsO_2_ exposure.

**Figure 5:**
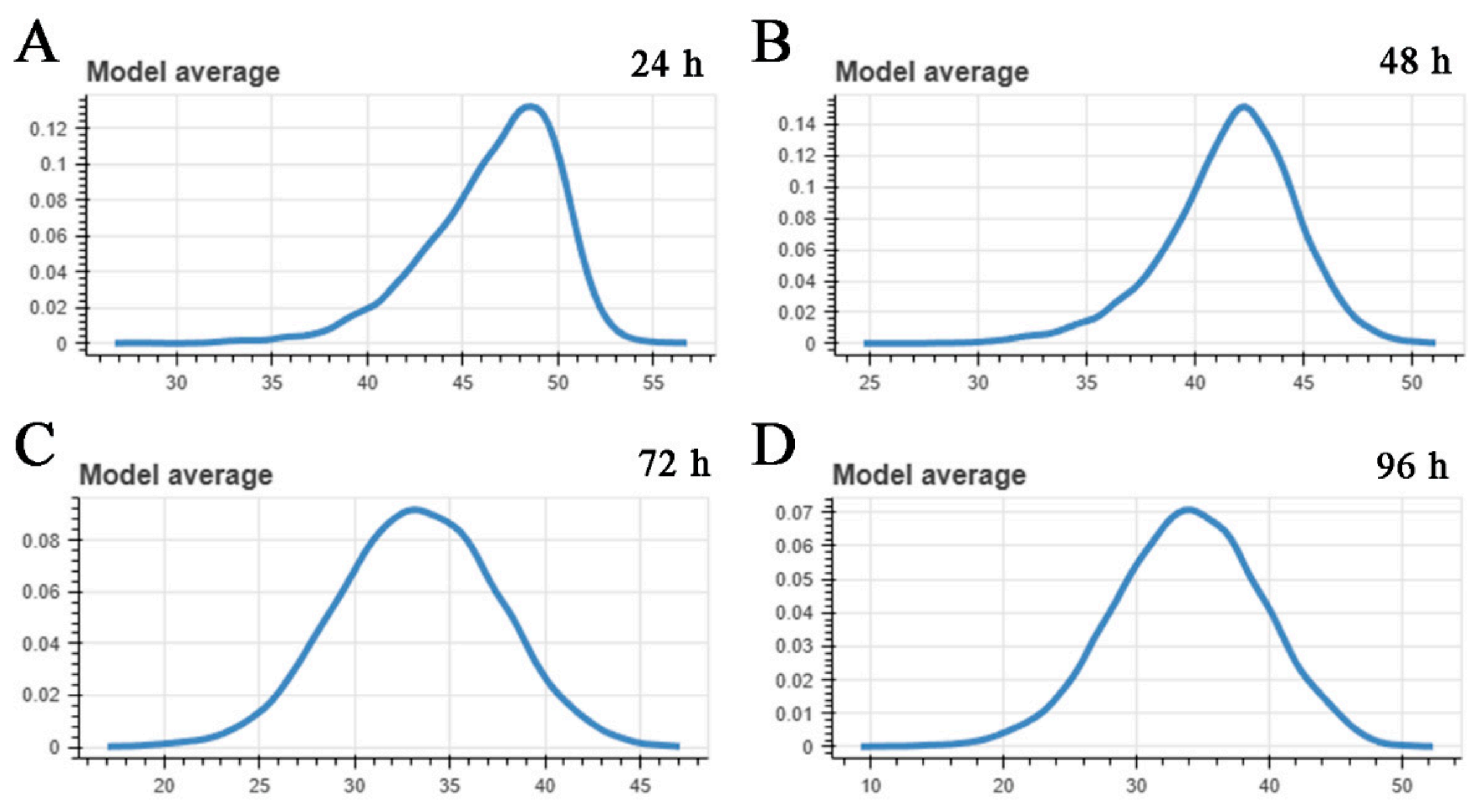
Posterior probability density of the BMD based on BMA in adult zebrafish after NaAsO_2_ exposure. (**A**) Posterior probability density of the BMD based on BMA in adult zebrafish after 24 h NaAsO_2_ exposure. (**B**) Posterior probability density of the BMD based on BMA in adult zebrafish after 48 h NaAsO_2_ exposure. (**C**) Posterior probability density of the BMD based on BMA in adult zebrafish after 72 h NaAsO_2_ exposure. (**D**) Posterior probability density of the BMD based on BMA in adult zebrafish after 96 h NaAsO_2_ exposure.

**Table 1:** LC_50_, BMD, BMDL and BMDU values of zebrafish embryo after NaAsO_2_ exposure in the BMA model.

	LC_50_	BMD (mg/L)	BMDL (mg/L)	BMDU (mg/L)
Embryo-24 h	1132.83	222.33	163.47	435.77
Embryo-48 h	734.70	348.34	230.09	486.77
Embryo-72 h	362.09	321.54	304.77	329.11
Embryo-96 h	217.16	47.20	42.24	93.08

**Table 2:** LC_50_, BMD, BMDL and BMDU values of larval zebrafish after NaAsO_2_ exposure in BMA model.

	LC_50_	BMD (mg/L)	BMDL (mg/L)	BMDU (mg/L)
Larva-24 h	118.30	34.53	12.91	109.34
Larva-48 h	108.71	61.28	16.95	97.96
Larva-72 h	103.30	60.24	33.61	84.39
Larva-96 h	92.17	76.76	45.76	86.81

**Table 3:** LC_50_, BMD, BMDL and BMDU values of juvenile zebrafish after NaAsO_2_ exposure in BMA model.

	LC_50_	BMD (mg/L)	BMDL (mg/L)	BMDU (mg/L)
Juvenile-24 h	84.81	47.47	19.18	59.27
Juvenile-48 h	71.20	42.19	34.12	49.03
Juvenile-72 h	70.98	38.77	31.72	44.90
Juvenile-96 h	64.12	41.72	33.88	48.66

**Table 4:** LC_50_, BMD, BMDL and BMDU values of adult zebrafish after NaAsO_2_ exposure in BMA model.

	LC_50_	BMD (mg/L)	BMDL (mg/L)	BMDU (mg/L)
Adult-24 h	71.96	47.22	40.16	51.13
Adult-48 h	57.90	41.95	36.15	46.13
Adult-72 h	56.13	33.32	26.32	40.13
Adult-96 h	54.93	33.95	24.49	42.90

## Data Availability

The original contributions presented in this study are included in the article/Supplementary Material. Further inquiries can be directed to the corresponding authors.

## Supplementary Materials

The following supporting information can be downloaded at https://www.mdpi.com/article/10.3390/toxics14100840/s1. Figure S1: Posterior probability density of the BMD based on eight BMD models in zebrafish embryo after NaAsO_2_ exposure; Figure S2: Dose-response relationship for eight BMD models in zebrafish embryo after NaAsO_2_ exposure; Figure S3: Posterior probability density of the BMD based on eight BMD models in larva zebrafish after NaAsO_2_ exposure; Figure S4: Dose-response relationship for eight BMD models in larva zebrafish after NaAsO_2_ exposure; Figure S5: Posterior probability density of the BMD based on eight BMD models in juvenile zebrafish after NaAsO_2_ exposure; Figure S6: Dose-response relationship for eight BMD models in juvenile zebrafish after NaAsO_2_ exposure; Figure S7: Posterior probability density of the BMD based on eight BMD models in adult zebrafish after NaAsO_2_ exposure; Figure S8: Dose-response relationship for eight BMD models in adult zebrafish after NaAsO_2_ exposure; Table S1: LC_50_, BMD, BMDL and BMDU values of eight different BMD models after 24 h NaAsO_2_ exposure in zebrafish embryo; Table S2: LC_50_, BMD, BMDL and BMDU values of eight different BMD models after 48 h NaAsO_2_ exposure in zebrafish embryo; Table S3: LC_50_, BMD, BMDL and BMDU values of eight different BMD models after 72 h NaAsO_2_ exposure in zebrafish embryo; Table S4: LC_50_, BMD, BMDL and BMDU values of eight different BMD models after 96 h NaAsO_2_ exposure in zebrafish embryo; Table S5: LC_50_, BMD, BMDL and BMDU values of eight different BMD models after NaAsO_2_ exposure in 24 h larva zebrafish; Table S6: LC_50_, BMD, BMDL and BMDU values of eight different BMD models after NaAsO_2_ exposure in 48 h larva zebrafish; Table S7: LC_50_, BMD, BMDL and BMDU values of eight different BMD models after NaAsO_2_ exposure in 72 h larva zebrafish; Table S8: LC_50_, BMD, BMDL and BMDU values of eight different BMD models after NaAsO_2_ exposure in 96 h larva zebrafish; Table S9: LC_50_, BMD, BMDL and BMDU values of eight different BMD models after NaAsO_2_ exposure in 24 h juvenile zebrafish; Table S10: LC_50_, BMD, BMDL and BMDU values of eight different BMD models after NaAsO_2_ exposure in 48 h juvenile zebrafish; Table S11: LC_50_, BMD, BMDL and BMDU values of eight different BMD models after NaAsO_2_ exposure in 72 h juvenile zebrafish; Table S12: LC_50_, BMD, BMDL and BMDU values of eight different BMD models after NaAsO_2_ exposure in 96 h juvenile zebrafish; Table S13: LC_50_, BMD, BMDL and BMDU values of eight different BMD models after NaAsO_2_ exposure in 24 h adult zebrafish; Table S14: LC_50_, BMD, BMDL and BMDU values of eight different BMD models after NaAsO_2_ exposure in 48 h adult zebrafish; Table S15: LC_50_, BMD, BMDL and BMDU values of eight different BMD models after NaAsO_2_ exposure in 72 h adult zebrafish; Table S16: LC_50_, BMD, BMDL and BMDU values of eight different BMD models after NaAsO_2_ exposure in 96 h adult zebrafish.

## Abbreviations

The following abbreviations are used in this manuscript:

BBMD	Bayesian Benchmark Dose Analysis System
BMA	Bayesian Model Averaging
BMD	Benchmark Dose
BMDL	Benchmark Dose Lower Bound
BMDU	Benchmark Dose Upper Bound
BMR	Benchmark Response
dpf	days post-fertilization
EPA	Environmental Protection Agency
EFSA	European Food Safety Authority
hpf	hours post-fertilization
LC_50_	median lethal dose
NOAEL	no observed adverse effect level
WHO	World Health Organization
