# Supplementary material for: Analysis of Arsenite Acute Toxic Effects Based on Bayesian Model Averaging in Embryo, Larva, Juvenile and Adult Zebrafish
Source: Toxics. 2026 Sep 22;14(10):840. doi: 10.3390/toxics14100840 (PMC13709699; doi:10.3390/toxics14100840)
Supplement: Supplementary file 1 [file toxics-14-00840-s001.zip › Supplementary Materials.pdf]

Supplementary Materials

Supplementary Table S1. LC_{50}, BMD, BMDL and BMDU values of eight different BMD models after 24 h $NaAsO_2$ exposure in zebrafish embryo.

Embryo-24 h	Model weight (%)	*PPP* value	LC_{50} (mg/L)	BMD (mg/L)	BMDL (mg/L)	BMDU (mg/L)
Exponential 2	5.55	0.573	1176.00	156.08	137.65	180.16
Exponential 3	21.47	0.534	11160.00	338.47	225.84	469.75
Exponential 4	0.47	0.534	1212.00	148.90	125.71	175.67
Exponential 5	4.71	0.523	1116.00	383.54	260.13	528.59
Hill	4.18	0.531	1128.00	341.04	218.55	489.43
Power	13.24	0.536	1116.00	314.97	226.12	450.28
Michaelis-Menten	2.38	0.528	1164.00	171.12	145.56	196.98
**Linear**	**47.99**	**0.534**	**1140.00**	**211.62**	**196.06**	**232.00**

Notes: The bolded value is the weight of the model with the highest weight proportion.

Supplementary Table S2. LC_{50}, BMD, BMDL and BMDU values of eight different BMD models after 48 h $NaAsO_2$ exposure in zebrafish embryo.

Embryo-48 h	Model weight (%)	*PPP* value	LC_{50} (mg/L)	BMD (mg/L)	BMDL (mg/L)	BMDU (mg/L)
Exponential 2	0.00	0.54	600.00	68.99	58.70	84.08
Exponential 3	15.16	0.54	730.00	448.84	340.58	563.66
Exponential 4	0.00	0.53	600.00	67.63	56.04	83.73
Exponential 5	1.48	0.53	730.00	467.72	339.88	626.65
Hill	13.75	0.54	730.00	359.17	253.85	483.32
**Power**	**66.73**	**0.53**	**740.00**	**339.91**	**227.26**	**464.04**
Michaelis-Menten	0.01	0.53	630.00	94.97	85.27	104.61
Linear	2.88	0.53	660.00	119.53	115.17	125.37

Notes: The bolded value is the weight of the model with the highest weight proportion.

Supplementary Table S3. LC_{50}, BMD, BMDL and BMDU values of eight different BMD models after 72 h $NaAsO_2$ exposure in zebrafish embryo.

Embryo-72 h	Model weight (%)	*PPP* value	LC_{50} (mg/L)	BMD (mg/L)	BMDL (mg/L)	BMDU (mg/L)
Exponential 2	0.00	0.54	412.50	53.00	37.37	90.67
Exponential 3	15.20	0.54	360.00	316.41	303.65	321.56
Exponential 4	0.00	0.53	550.00	40.70	1.93	94.61
Exponential 5	1.94	0.53	360.00	314.68	298.02	321.00
Hill	13.19	0.54	360.00	318.79	299.54	325.53
**Power**	**69.67**	**0.54**	**363.00**	**322.47**	**305.14**	**329.26**
Michaelis-Menten	0.00	0.53	375.00	67.69	45.67	127.44
Linear	0.00	0.54	371.30	66.28	54.78	93.15

Notes: The bolded value is the weight of the model with the highest weight proportion.

Supplementary Table S4. LC_{50}, BMD, BMDL and BMDU values of eight different BMD models after 96 h $NaAsO_2$ exposure in zebrafish embryo.

Embryo-96 h	Model weight (%)	*PPP* value	LC_{50} (mg/L)	BMD (mg/L)	BMDL (mg/L)	BMDU (mg/L)
Exponential 2	6.91	0.56	177.00	31.66	27.09	37.88
Exponential 3	11.17	0.55	222.00	89.46	47.21	139.70
Exponential 4	0.82	0.55	198.00	29.17	21.05	36.52
Exponential 5	1.81	0.55	216.00	104.09	49.21	167.36
Hill	3.49	0.55	219.00	73.98	42.24	131.38
Power	15.15	0.55	228.00	74.37	49.54	124.23
Michaelis-Menten	6.86	0.56	213.00	38.37	31.50	45.16
**Linear**	**53.79**	**0.54**	**219.00**	**46.80**	**43.49**	**51.53**

Notes: The bolded value is the weight of the model with the highest weight proportion.

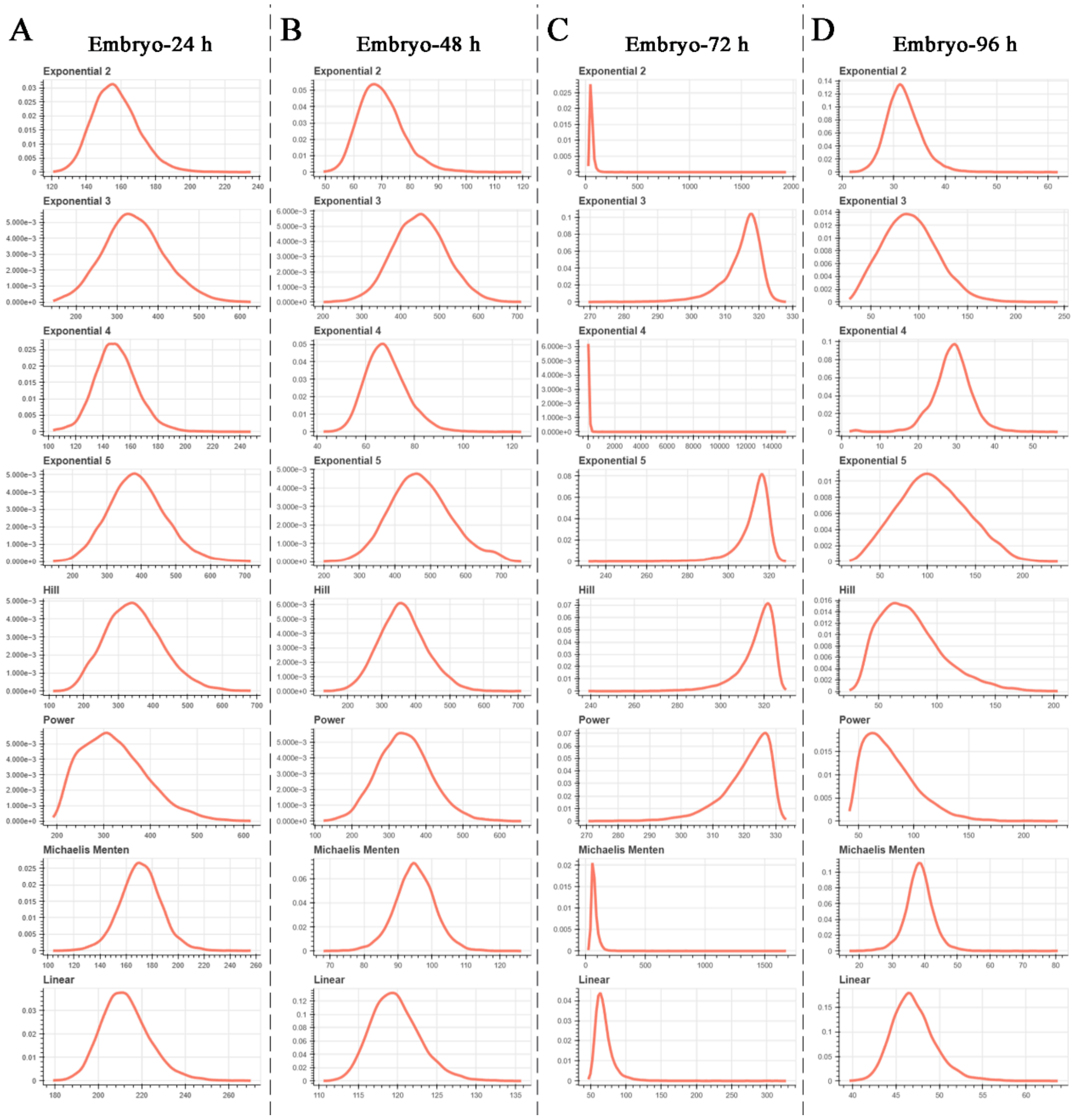

Supplementary Figure S1. Posterior probability density of the BMD based on eight BMD models in zebrafish embryo after $NaAsO_2$ exposure.

(A) Posterior probability density of the BMD based on eight BMD models in zebrafish embryo after 24 h $NaAsO_2$ exposure. (B) Posterior probability density of the BMD based on eight BMD models in zebrafish embryo after 48 h $NaAsO_2$ exposure. (C) Posterior probability density of the BMD based on eight BMD models in zebrafish embryo after 72 h $NaAsO_2$ exposure. (D) Posterior probability density of the BMD based on eight BMD models in zebrafish embryo after 96 h $NaAsO_2$ exposure.

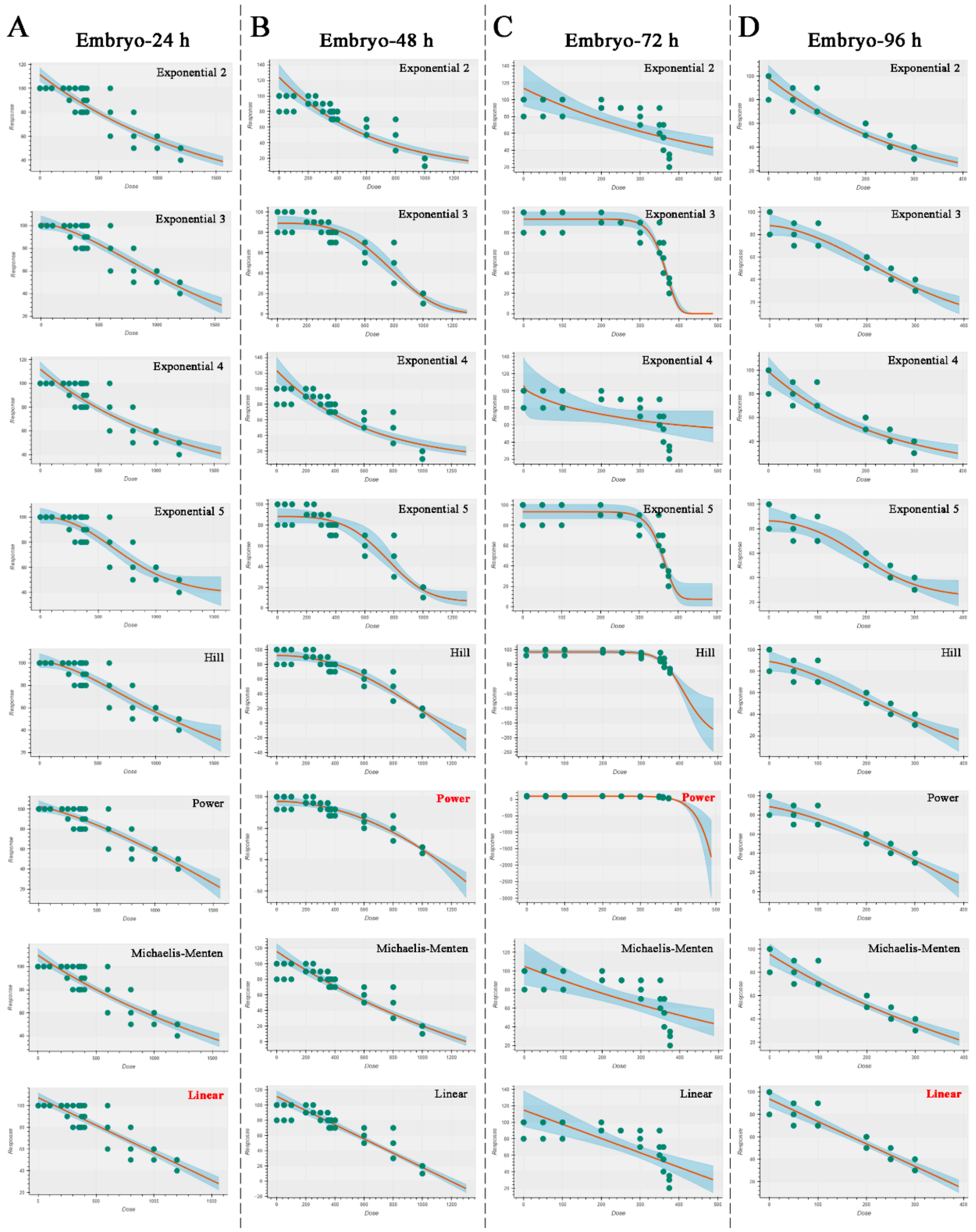

Supplementary Figure S2. Dose-response relationship for eight BMD models in zebrafish embryo after $NaAsO_2$ exposure. (A) Dose-response relationship for eight BMD models in zebrafish embryo after 24 h $NaAsO_2$ exposure. (B) Dose-response relationship for eight BMD models in zebrafish embryo after 48 h $NaAsO_2$ exposure. (C) Dose-response relationship for eight BMD models in zebrafish embryo after 72 h $NaAsO_2$ exposure. (D) Dose-response relationship for eight BMD models in zebrafish embryo after 96 h $NaAsO_2$ exposure.

Notes: The red-colored model is the weight of the model with the highest weight proportion.

Supplementary Table S5. LC_{50}, BMD, BMDL and BMDU values of eight different BMD models after $NaAsO_2$ exposure in 24 h larva zebrafish.

Larva-24 h	Model weight (%)	*PPP* value	LC_{50} (mg/L)	BMD (mg/L)	BMDL (mg/L)	BMDU (mg/L)
Exponential 2	8.81	0.55	94.00	10.21	8.17	13.75
Exponential 3	16.01	0.55	120.00	46.29	19.57	86.67
Exponential 4	0.90	0.56	88.00	9.32	5.06	13.83
Exponential 5	20.61	0.54	120.00	90.75	48.23	118.43
Hill	17.22	0.54	120.00	81.06	22.14	110.56
Power	3.69	0.55	134.00	43.90	24.58	122.25
Michaelis-Menten	7.89	0.55	112.00	18.42	12.91	22.39
**Linear**	**24.87**	**0.55**	**124.00**	**23.00**	**21.72**	**25.59**

Notes: The bolded value is the weight of the model with the highest weight proportion.

Supplementary Table S6. LC_{50}, BMD, BMDL and BMDU values of eight different BMD models after $NaAsO_2$ exposure in 48 h larva zebrafish.

Larva-48 h	Model weight (%)	*PPP* value	LC_{50} (mg/L)	BMD (mg/L)	BMDL (mg/L)	BMDU (mg/L)
Exponential 2	0.01	0.54	75.00	8.07	6.40	11.07
**Exponential 3**	**34.64**	**0.56**	**109.50**	**64.25**	**44.41**	**98.28**
Exponential 4	0.00	0.55	72.00	7.52	1.28	11.89
Exponential 5	14.34	0.54	103.50	79.87	54.59	92.20
Hill	12.15	0.56	109.50	58.47	30.36	89.96
Power	25.36	0.54	115.50	55.01	28.27	112.27
Michaelis-Menten	0.32	0.55	90.00	12.66	10.19	14.84
Linear	13.18	0.55	99.00	16.89	16.31	17.81

Notes: The bolded value is the weight of the model with the highest weight proportion.

Supplementary Table S7. LC_{50}, BMD, BMDL and BMDU values of eight different BMD models after $NaAsO_2$ exposure in 72 h larva zebrafish.

Larva-72 h	Model weight (%)	*PPP* value	LC_{50} (mg/L)	BMD (mg/L)	BMDL (mg/L)	BMDU (mg/L)
Exponential 2	0.00	0.55	66.00	7.01	5.83	8.90
Exponential 3	28.14	0.55	94.50	44.24	34.75	54.99
Exponential 4	0.00	0.55	63.00	6.71	4.76	9.00
**Exponential 5**	**39.17**	**0.56**	**96.50**	**72.70**	**48.20**	**85.61**
Hill	27.68	0.57	95.00	63.36	29.38	82.55
Power	1.55	0.56	99.00	28.45	18.34	48.04
Michaelis-Menten	0.01	0.55	90.00	12.33	10.54	13.39
Linear	13.18	0.55	84.00	16.47	16.15	16.89

Notes: The bolded value is the weight of the model with the highest weight proportion.

Supplementary Table S8. LC_{50}, BMD, BMDL and BMDU values of eight different BMD models after $NaAsO_2$ exposure in 96 h larva zebrafish.

Larva-96 h	Model weight (%)	*PPP* value	LC_{50} (mg/L)	BMD (mg/L)	BMDL (mg/L)	BMDU (mg/L)
Exponential 2	0.25	0.56	77.00	9.25	6.63	15.08
Exponential 3	35.86	0.56	94.00	76.30	47.35	84.80
Exponential 4	0.02	0.55	140.00	5.63	0.32	15.25
Exponential 5	12.43	0.56	77.00	58.23	43.44	79.14
Hill	12.39	0.55	92.00	66.07	36.89	84.61
**Power**	**36.33**	**0.57**	**96.50**	**80.19**	**47.77**	**87.52**
Michaelis-Menten	0.18	0.56	81.00	12.10	8.53	23.58
Linear	2.55	0.56	81.00	14.25	12.64	17.69

Notes: The bolded value is the weight of the model with the highest weight proportion.

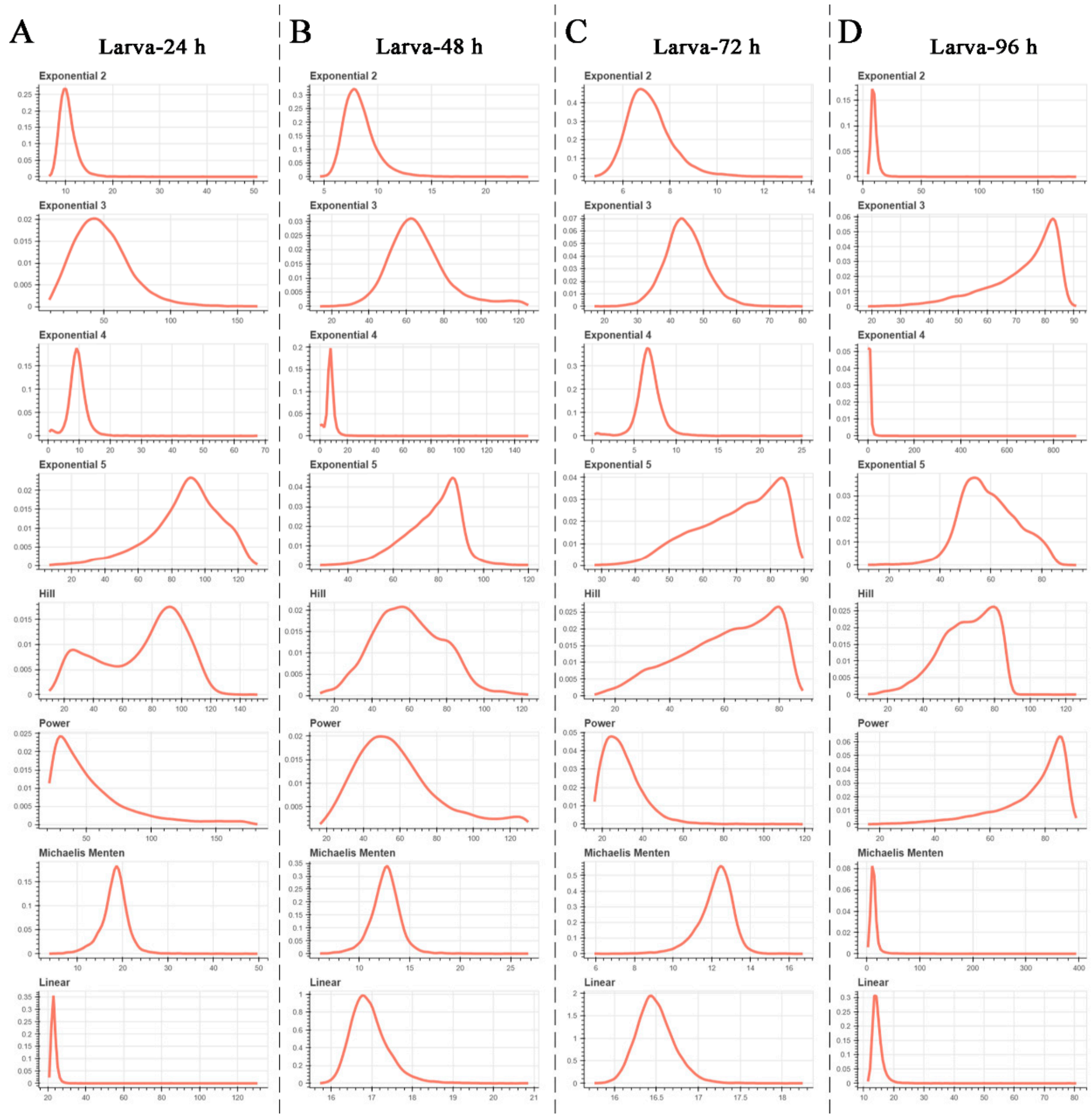

Supplementary Figure S3. Posterior probability density of the BMD based on eight BMD models in larva zebrafish after $NaAsO_2$ exposure.

(A) Posterior probability density of the BMD based on eight BMD models in larva zebrafish after 24 h $NaAsO_2$ exposure. (B) Posterior probability density of the BMD based on eight BMD models in larva zebrafish after 48 h $NaAsO_2$ exposure. (C) Posterior probability density of the BMD based on eight BMD models in larva zebrafish after 72 h $NaAsO_2$ exposure. (D) Posterior probability density of the BMD based on eight BMD models in larva zebrafish after 96 h $NaAsO_2$ exposure.

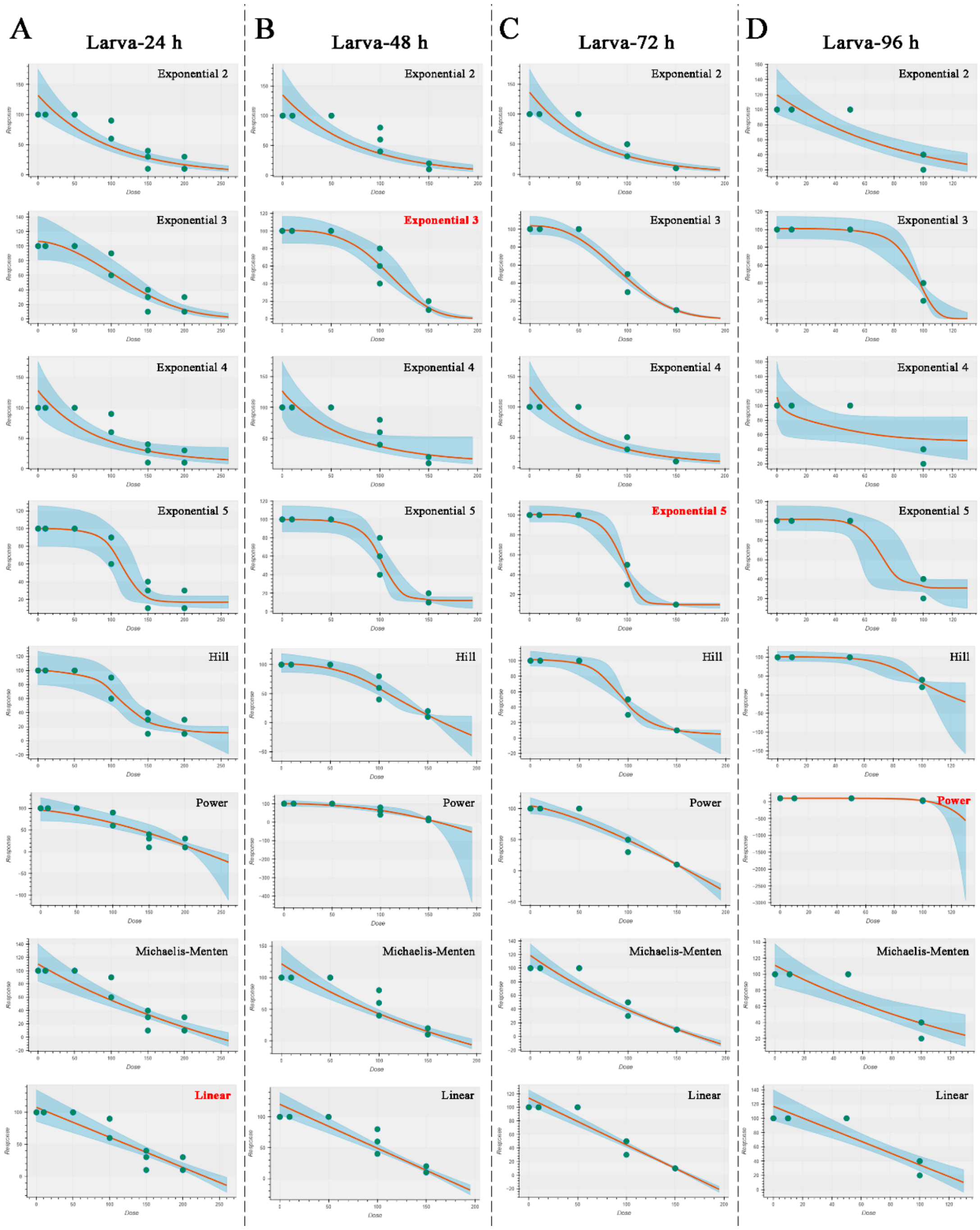

Supplementary Figure S4. Dose-response relationship for eight BMD models in larva zebrafish after $NaAsO_2$ exposure. (A) Dose-response relationship for eight BMD models in larva zebrafish after 24 h $NaAsO_2$ exposure. (B) Dose-response relationship for eight BMD models in larva zebrafish after 48 h $NaAsO_2$ exposure. (C) Dose-response relationship for eight BMD models in larva zebrafish after 72 h $NaAsO_2$ exposure. (D) Dose-response relationship for eight BMD models in larva zebrafish after 96 h $NaAsO_2$ exposure.

Notes: The red-colored model is the weight of the model with the highest weight proportion.

Supplementary Table S9. LC_{50}, BMD, BMDL and BMDU values of eight different BMD models after $NaAsO_2$ exposure in 24 h juvenile zebrafish.

Juvenile-24 h	Model weight (%)	*PPP* value	LC_{50} (mg/L)	BMD (mg/L)	BMDL (mg/L)	BMDU (mg/L)
Exponential 2	5.87	0.55	100.00	16.97	12.20	27.62
**Exponential 3**	**31.49**	**0.55**	**77.00**	**48.78**	**34.17**	**59.54**
Exponential 4	0.49	0.55	100.00	13.54	0.52	26.76
Exponential 5	10.01	0.54	100.00	49.13	33.90	57.48
Hill	9.36	0.55	84.00	46.85	27.91	57.30
Power	30.25	0.55	76.30	47.89	32.27	59.91
Michaelis-Menten	1.12	0.55	103.00	20.05	12.98	39.10
Linear	11.41	0.55	106.00	19.17	15.11	28.08

Notes: The bolded value is the weight of the model with the highest weight proportion.

Supplementary Table S10. LC_{50}, BMD, BMDL and BMDU values of eight different BMD models after $NaAsO_2$ exposure in 48 h juvenile zebrafish.

Juvenile-48 h	Model weight (%)	*PPP* value	LC_{50} (mg/L)	BMD (mg/L)	BMDL (mg/L)	BMDU (mg/L)
Exponential 2	0.00	0.55	92.00	12.35	9.81	16.53
Exponential 3	35.98	0.55	71.40	42.83	35.17	49.39
Exponential 4	0.00	0.55	95.00	11.65	8.29	16.46
Exponential 5	7.71	0.53	72.10	43.16	35.50	49.42
Hill	10.58	0.55	72.00	42.49	34.32	49.06
**Power**	**45.68**	**0.55**	**70.70**	**41.59**	**33.38**	**48.75**
Michaelis-Menten	0.00	0.55	95.00	13.30	10.02	18.96
Linear	0.05	0.54	82.60	14.86	12.94	17.78

Notes: The bolded value is the weight of the model with the highest weight proportion.

Supplementary Table S11. LC_{50}, BMD, BMDL and BMDU values of eight different BMD models after $NaAsO_2$ exposure in 72 h juvenile zebrafish.

Juvenile-72 h	Model weight (%)	*PPP* value	LC_{50} (mg/L)	BMD (mg/L)	BMDL (mg/L)	BMDU (mg/L)
Exponential 2	0.00	0.54	90.00	11.64	9.53	14.92
Exponential 3	38.55	0.55	70.70	39.55	33.09	45.29
Exponential 4	0.00	0.53	94.00	11.06	8.43	14.99
Exponential 5	9.77	0.56	71.40	40.57	34.09	46.01
Hill	11.74	0.54	71.70	39.63	32.70	45.54
**Power**	**39.93**	**0.55**	**70.70**	**37.71**	**30.81**	**44.05**
Michaelis-Menten	0.00	0.55	95.00	12.41	9.76	16.71
Linear	0.13	0.54	79.10	14.20	12.65	16.45

Notes: The bolded value is the weight of the model with the highest weight proportion.

Supplementary Table S12. LC_{50}, BMD, BMDL and BMDU values of eight different BMD models after $NaAsO_2$ exposure in 96 h juvenile zebrafish.

Juvenile-96 h	Model weight (%)	*PPP* value	LC_{50} (mg/L)	BMD (mg/L)	BMDL (mg/L)	BMDU (mg/L)
Exponential 2	0.00	0.54	65.10	7.58	5.82	10.80
Exponential 3	23.13	0.54	63.70	44.12	37.58	50.51
Exponential 4	0.00	0.54	69.30	6.90	0.49	11.09
Exponential 5	3.57	0.55	63.70	44.33	37.15	50.71
Hill	12.38	0.54	63.70	42.42	35.16	48.84
**Power**	**60.91**	**0.54**	**64.40**	**41.17**	**33.45**	**47.92**
Michaelis-Menten	0.00	0.56	65.80	8.99	6.66	13.31
Linear	0.00	0.54	62.30	10.36	9.29	12.17

Notes: The bolded value is the weight of the model with the highest weight proportion.

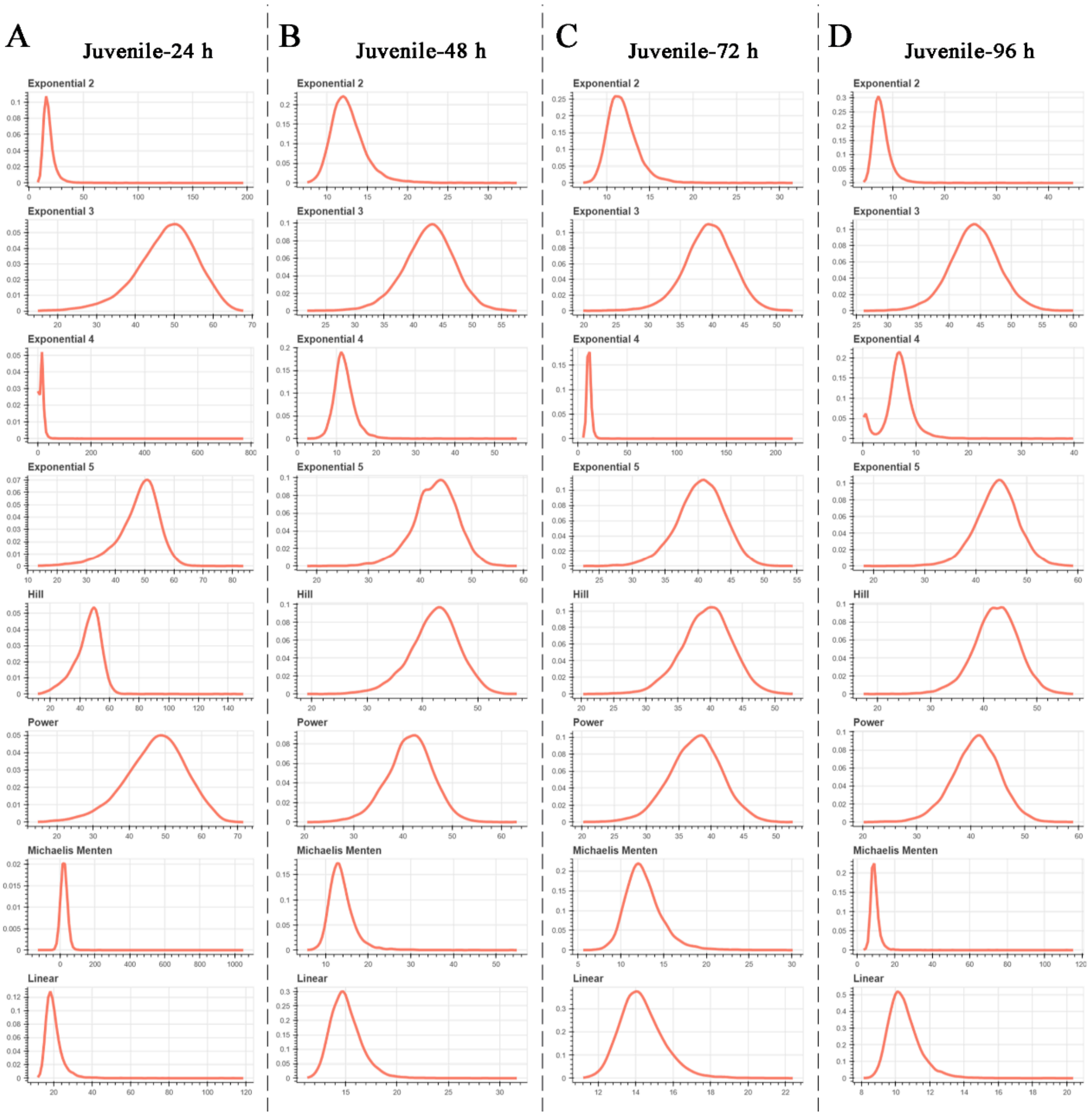

Supplementary Figure S5. Posterior probability density of the BMD based on eight BMD models in juvenile zebrafish after $NaAsO_2$ exposure.

(A) Posterior probability density of the BMD based on eight BMD models in juvenile zebrafish after 24 h $NaAsO_2$ exposure. (B) Posterior probability density of the BMD based on eight BMD models in juvenile zebrafish after 48 h $NaAsO_2$ exposure. (C) Posterior probability density of the BMD based on eight BMD models in juvenile zebrafish after 72 h $NaAsO_2$ exposure. (D) Posterior probability density of the BMD based on eight BMD models in juvenile zebrafish after 96 h $NaAsO_2$ exposure.

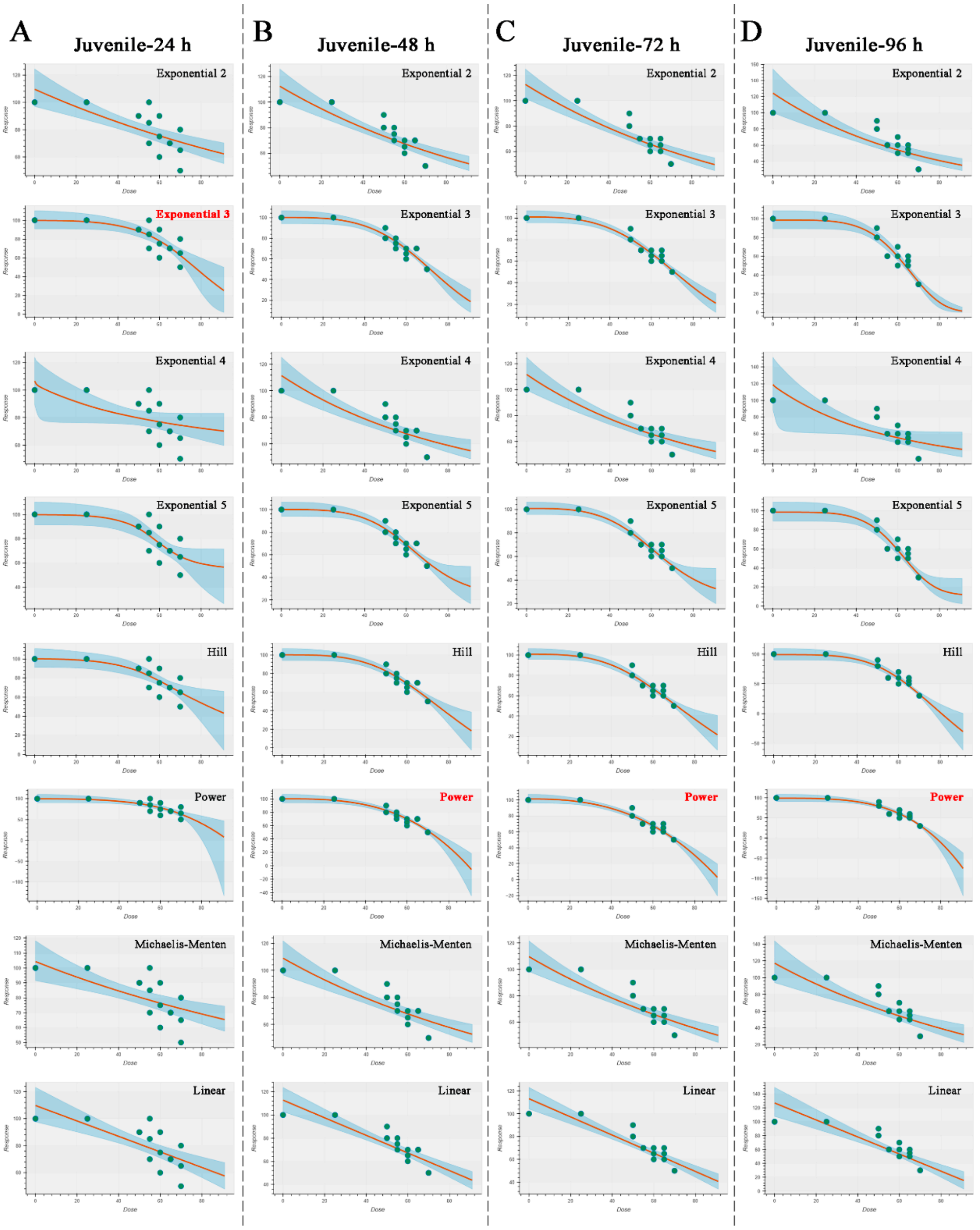

Supplementary Figure S6. Dose-response relationship for eight BMD models in juvenile zebrafish after $NaAsO_2$ exposure. (A) Dose-response relationship for eight BMD models in juvenile zebrafish after 24 h $NaAsO_2$ exposure. (B) Dose-response relationship for eight BMD models in juvenile zebrafish after 48 h $NaAsO_2$ exposure. (C) Dose-response relationship for eight BMD models in juvenile zebrafish after 72 h $NaAsO_2$ exposure. (D) Dose-response relationship for eight BMD models in juvenile zebrafish after 96 h $NaAsO_2$ exposure.

Notes: The red-colored model is the weight of the model with the highest weight proportion.

Supplementary Table S13. LC_{50}, BMD, BMDL and BMDU values of eight different BMD models after $NaAsO_2$ exposure in 24 h adult zebrafish.

Adult-24 h	Model weight (%)	*PPP* value	LC_{50} (mg/L)	BMD (mg/L)	BMDL (mg/L)	BMDU (mg/L)
Exponential 2	0.00	0.54	91.00	11.21	8.19	17.24
Exponential 3	27.50	0.53	65.00	45.64	39.29	50.82
Exponential 4	0.00	0.55	91.00	10.87	6.75	19.61
**Exponential 5**	**33.83**	**0.53**	**85.20**	**48.47**	**43.20**	**51.38**
Hill	22.72	0.53	65.60	47.26	40.47	50.91
Power	15.94	0.54	65.00	44.35	36.76	50.62
Michaelis-Menten	0.00	0.53	95.00	13.14	9.01	25.12
Linear	0.00	0.54	77.35	13.00	10.91	17.10

Notes: The bolded value is the weight of the model with the highest weight proportion.

Supplementary Table S14. LC_{50}, BMD, BMDL and BMDU values of eight different BMD models after $NaAsO_2$ exposure in 48 h adult zebrafish.

Adult-48 h	Model weight (%)	*PPP* value	LC_{50} (mg/L)	BMD (mg/L)	BMDL (mg/L)	BMDU (mg/L)
Exponential 2	0.00	0.54	59.00	5.80	4.48	8.49
**Exponential 3**	**53.56**	**0.53**	**57.80**	**42.24**	**38.11**	**45.95**
Exponential 4	0.00	0.53	50.60	5.54	0.43	9.28
Exponential 5	14.40	0.54	57.50	43.69	39.36	47.73
Hill	12.92	0.54	57.85	41.80	35.99	47.02
Power	19.12	0.54	58.50	38.00	32.27	43.32
Michaelis-Menten	0.00	0.53	50.20	7.01	5.31	10.07
Linear	0.00	0.54	55.90	8.34	7.72	9.41

Notes: The bolded value is the weight of the model with the highest weight proportion.

Supplementary Table S15. LC_{50}, BMD, BMDL and BMDU values of eight different BMD models after $NaAsO_2$ exposure in 72 h adult zebrafish.

Adult-72 h	Model weight (%)	*PPP* value	LC_{50} (mg/L)	BMD (mg/L)	BMDL (mg/L)	BMDU (mg/L)
Exponential 2	0.00	0.53	53.90	5.56	4.48	7.38
Exponential 3	26.07	0.54	55.90	37.22	31.68	42.22
Exponential 4	0.00	0.54	50.70	5.40	4.10	7.55
Exponential 5	4.03	0.54	55.90	38.02	32.20	43.55
Hill	13.11	0.54	55.90	34.54	28.28	40.52
**Power**	**56.79**	**0.54**	**56.30**	**32.13**	**25.73**	**38.10**
Michaelis-Menten	0.00	0.53	53.30	6.81	5.56	8.59
Linear	0.00	0.54	53.30	8.47	7.96	9.25

Notes: The bolded value is the weight of the model with the highest weight proportion.

Supplementary Table S16. LC_{50}, BMD, BMDL and BMDU values of eight different BMD models after $NaAsO_2$ exposure in 96 h adult zebrafish.

Adult-96 h	Model weight (%)	*PPP* value	LC_{50} (mg/L)	BMD (mg/L)	BMDL (mg/L)	BMDU (mg/L)
Exponential 2	0.00	0.54	50.60	4.77	3.84	6.53
Exponential 3	24.20	0.54	54.60	38.97	31.66	45.41
Exponential 4	0.00	0.53	50.40	4.68	3.36	7.00
Exponential 5	3.50	0.54	54.20	39.60	31.87	46.57
Hill	13.75	0.54	54.60	34.97	26.66	42.56
**Power**	**58.48**	**0.53**	**55.20**	**32.62**	**23.74**	**40.80**
Michaelis-Menten	0.00	0.54	50.10	6.21	4.92	7.98
Linear	0.07	0.53	51.30	7.83	7.40	8.53

Notes: The bolded value is the weight of the model with the highest weight proportion.

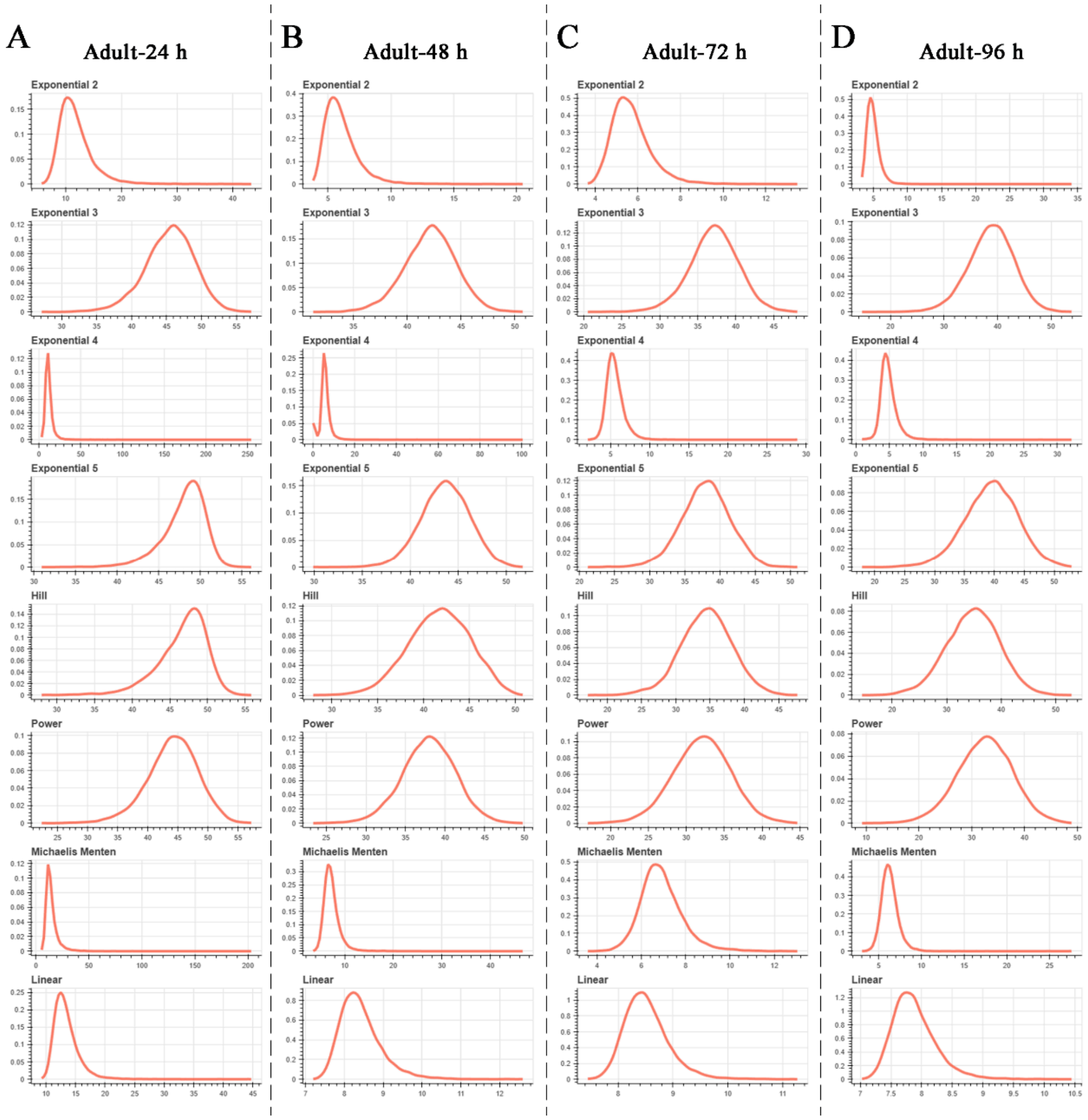

Supplementary Figure S7. Posterior probability density of the BMD based on eight BMD models in adult zebrafish after $NaAsO_2$ exposure.

(A) Posterior probability density of the BMD based on eight BMD models in adult zebrafish after 24 h $NaAsO_2$ exposure. (B) Posterior probability density of the BMD based on eight BMD models in adult zebrafish after 48 h $NaAsO_2$ exposure. (C) Posterior probability density of the BMD based on eight BMD models in adult zebrafish after 72 h $NaAsO_2$ exposure. (D) Posterior probability density of the BMD based on eight BMD models in adult zebrafish after 96 h $NaAsO_2$ exposure.

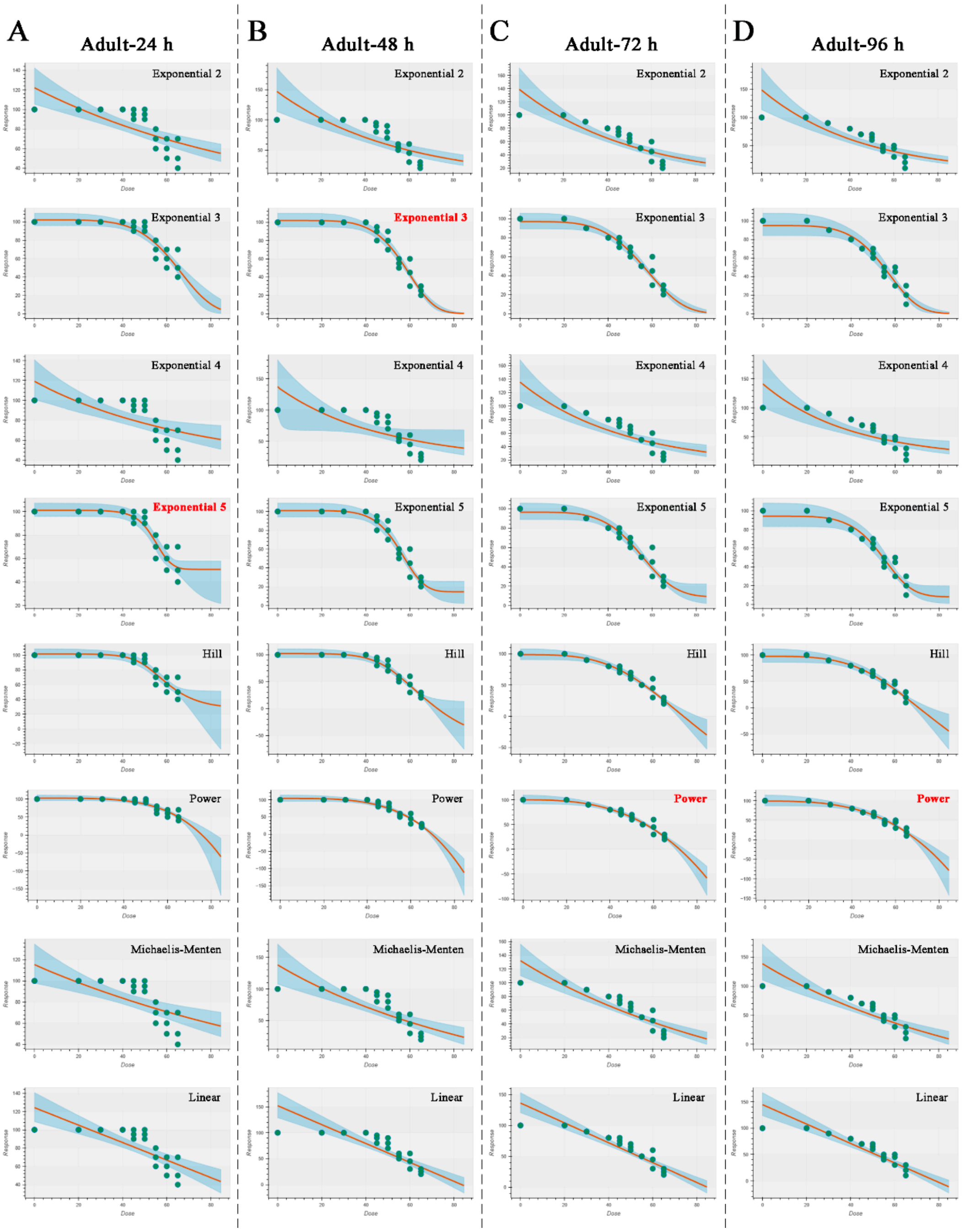

Supplementary Figure S8. Dose-response relationship for eight BMD models in adult zebrafish after $NaAsO_2$ exposure. (A) Dose-response relationship for eight BMD models in adult zebrafish after 24 h $NaAsO_2$ exposure. (B) Dose-response relationship for eight BMD models in adult zebrafish after 48 h $NaAsO_2$ exposure. (C) Dose-response relationship for eight BMD models in adult zebrafish after 72 h $NaAsO_2$ exposure. (D) Dose-response relationship for eight BMD models in adult zebrafish after 96 h $NaAsO_2$ exposure.

Notes: The red-colored model is the weight of the model with the highest weight proportion.
